# Laboratory chemical waste: hazard classification by GHS and transport risk

**DOI:** 10.11606/s1518-8787.2021055003259

**Published:** 2021-11-23

**Authors:** Cássio Giovanni, Fabio Luiz Navarro Marques, Wanda Maria Risso Günther

**Affiliations:** I Universidade de São Paulo Faculdade de Saúde Pública Programa de Pós-Graduação em Saúde Global e Sustentabilidade São Paulo SP Brasil Universidade de São Paulo. Faculdade de Saúde Pública. Programa de Pós-Graduação em Saúde Global e Sustentabilidade. São Paulo, SP, Brasil; II Universidade de São Paulo Faculdade de Medicina Departamento de Radiologia São Paulo SP Brasil Universidade de São Paulo. Faculdade de Medicina. Departamento de Radiologia. São Paulo, SP, Brasil; III Universidade de São Paulo Faculdade de Saúde Pública Departamento de Saúde Ambiental São Paulo SP Brasil Universidade de São Paulo. Faculdade de Saúde Pública. Departamento de Saúde Ambiental. São Paulo, SP, Brasil

**Keywords:** Chemical Waste, Hazardous Waste, Product Labeling, Substances, Products and Materials Transportation

## Abstract

**OBJECTIVES:**

To identify and evaluate, based on the Globally Harmonized System of Classification and Labelling of Chemicals (GHS) and the legislation of the *Agência Nacional de Transportes Terrestres* (ANTT – National Agency for Terrestrial Transport), the hazards arising from chemical waste generated in research laboratories in the health area.

**METHODS:**

Chemical residues generated in two medical research laboratories of the Faculdade de Medicina da Universidade de São Paulo were inventoried, from November 2017 to April 2019, and classified according to the GHS (hazard statements) and the ANTT transport legislation (risk classes), to determine the dangers coming from the respective substances and mixtures.

**RESULTS:**

In total, we identified 40 substances or mixtures with classification by the GHS indicating 36 hazard statements, 27 of which related to human health. According to the legislation established by ANTT, we found 16 cases of hazard associated with flammability, 15 cases related to toxicity and 12 cases related to corrosivity.

**CONCLUSIONS:**

Chemical residues generated in the laboratories studied are diversified in terms of their hazard characteristics, implying the possibility of exposure to severe risks to workers, students and the environment. The correct identification of these residues is a primary factor for reducing exposure to risks.

## INTRODUCTION

The contemporary context of production processes and the economic model, with significant expansion of the consumption of chemicals, greater speed in the synthesis of new compounds and increased extraction of raw materials, potentiates the generation of waste of various categories.

Chemical products and waste in the environment can affect species and ecosystems and even cause large-scale problems such as stratospheric ozone depletion^1^. Chemical compounds, if used and/or discarded in disagreement with technical, scientific and legal guidelines, can change the characteristics and quality of environmental compartments (air, water, soil and biome), as well as cause damage to health, physical integrity and human activities.

The lack of knowledge about the properties and hazardousness of chemical products and waste may even result in occupational damage to professionals involved in the handling and/or management of such substances, mixtures or materials. Therefore, it is essential that workers and students involved in these activities be trained and oriented on the risks associated with chemicals, as well as those arising from the inadequate management of the waste generated.

A key aspect in this process is the creation of the Globally Harmonized System of Classification and Labelling of Chemicals (GHS), formally adopted in July 2003 by the United Nations Economic and Social Committee^2^. It is a system that promotes the identification and classification of dangerous chemicals in a standardized and harmonized way, using statements and pictograms affixed to the labels of the packaging and informed in safety sheets about the substance, mixture or material in question.

In Brazil, the classification and preventive labeling of chemicals through the GHS are mandatory, since the adoption of the system is provided for in Regulatory Standard (RS) No. 26 – Safety Signaling, since it was updated by Ordinance No. 229/2011, of the Labor Inspection Department. In addition, this update states that the structure and content of the Chemical Safety Data Sheet should follow GHS guidelines^3^. The application of GHS for classification, labeling and safety data sheets of chemical products is guided by the Brazilian standard (NBR) 14,725, Parts 1, 2, 3 and 4, of the *Associação Brasileira de Normas Técnicas* (ABNT)^4–7^.

**Figure 1:**
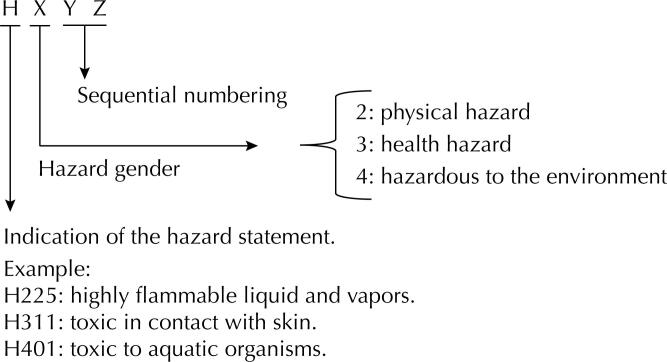
Specification of hazard statements (H) according to GHS.

Sources: Elaborated by the authors, based on ABNT^4^, UNECE^8^ and Wallau and Santos^9^.

One of the provisions of the GHS consists of hazard statements, which concern standardized texts describing the nature and, if appropriate, the severity of the hazard^7–9^. The configuration of hazard statements, indicated by the letter H (hazard), is explained in Figure 1.

The GHS proposes other elements for hazard communication, such as labeling, precautionary statements and safety data sheets^2,4,5^.

As Brazilian public institutions and private companies must meet the requirements regarding the application and precepts of the GHS, to ensure and improve the conditions of labor safety in their facilities, it becomes essential to train workers and students, with the purpose of eliminating or mitigating environmental impacts, health and labor safety.

From the perspective of external transport, hazardous products (and waste), whether of chemical, biological or radiological origin, represent potential risks to life, health, the environment and public safety^10^. To identify the hazard in the transport of hazardous cargoes, Resolution No. 5,947/2021, of the *Agência Nacional de Transportes Terrestres* (ANTT – National Agency for Land Transport) defined nine risk classes expressed by internationally standardized numbers^11^.

Hazard classes, their respective pictograms and identification codes (UN numbers) represent universal language for recognizing transported hazardous products and waste. Therefore, they are fundamental for taking action in the event of accidents, leaks or spills, or even for international cross-border transport.

In general, chemical waste from diverse segments such as industry, commerce, health services, education and research are treated outside the generating units. Thus, its management includes external steps and procedures, such as transportation to treatment or final disposal. In this case, according to Federal Laws No. 12,305/2010 (Articles 20 and 27) and No. 9,605/1998 (Articles 2, 3, 54 and 56), the generating establishment is co-responsible (together with the transporting agent) for properly classifying chemical waste within the scope of the displacement of hazardous cargoes, according to the risk classes^12,13^.

In identification and labeling, knowledge about risk classes and their legislation becomes essential for the correct completion of the waste transport manifest (WTM), a mandatory document in the stage of handling hazardous goods. The class or subclass of risks and, if existing, subsidiary risks are paramount to define the due incompatibilities between chemical residues, according to ABNT NBR 14,619/2021^14^.

The organization of such waste through the GHS and the transport system enables a systemic understanding of the associated chemical risks. Such risks are considered from the internal and external perspectives of the institution and the generating laboratory environment, in a continuous and complementary manner, considering the entire process of managing the chemical waste generated.

It is considered that many substances can be considered hazardous in the GHS and/or ANTT parameters, although they have not yet been studied and classified as potential hazards, especially on toxicity, mutagenicity, carcinogenicity and ecotoxicity. Therefore, when dealing with such substances, the precautionary principle must be adopted to safeguard human and environmental health.

This article aims to identify and evaluate, based on the GHS (hazard statements) and the ANTT transport legislation (risk classes), the chemical waste generated in research laboratories in the health area, contributing to the improvement of chemical waste and risk management and the adoption of precautionary, prevention and contingency measures. This study can support for the implementation of quality policy in institutions similar to the one studied, disseminating to all laboratories and units the bases to meet the relevant legislation for the management of chemical waste.

## METHODS

The research was conducted at the Faculdade de Medicina da São Paulo (FMUSP), in two Medical Research Laboratories (MRL), whose chemical residues generated are varied and complex, many of which consist of mixtures of substances. As a data source, a retrospective analysis of the chemical residues generated and declared by the laboratories in inventory sheets was carried out from November 2017 to April 2019. These data sheets are used to document the procedure for forwarding chemical waste from laboratories to the institution's external storage facility.

To facilitate and unify the understanding and identification of each chemical residue, the terminology of the items listed by the laboratories were standardized according to the technical and/or official nomenclature of substances. Furthermore, in the stage of determining the hazards arising from the chemical waste generated, the laboratory scope (GHS, ABNT NBR 14,725-3/2017) and the perspective of external transport (ANTT Resolution No. 5,947/2021) were considered together.

In the case of GHS, standardized hazard statements (ABNT NBR 14,725-3/2017) were assigned to chemical residues in order to identify intrinsic hazard characteristics of the substances present. Such statements (H – hazard) are represented by codes and are part of the formulation and application of GHS^6^. In addiction, for each component substance of the chemical residue, hazard statements were obtained through research on the portal of the European Chemicals Agency (ECHA)^15^.

In the classification of empty packaging and other objects that had contact with chemicals, the characteristics of contaminating substances or mixtures were considered. For residues containing mixtures of oxidizing and flammable substances, such as hydrogen peroxide and methanol, respectively, codes and hazard statements of flammable compounds were used, due to the low concentration of oxidants verified.

As for transportation, chemical waste was classified by its risk class or subclass and subsidiary risk, if any.

## RESULTS

In Laboratory I, we found 14 types of chemical waste (Table 1), listed in alphabetical order and classified according to the GHS (based on ABNT NBR 14,725-3/2017 and on the ECHA website) and ANTT Resolution No. 5,947/2021 (transport of hazardous goods).

**Table 1:** Declared relationship of chemical residues generated in FMUSP Laboratory I, in the period from November 2017 to April 2019.

Chemical residue composition	Hazard statements (GHS codes)	Class or subclass of risk and subsidiary risk (ANTT)
Acetone	H225, H319, H336	3
Hydrochloric acid, solution	H290, H314, H331	8
Picric acid	H201, H301, H311, H331	4.1
Beta-mercaptoethanol	H301, H310, H311, H315, H318, H331, H400, H411	6.1
Phenol	H301, H311, H314, H331, H341, H373, H411	6.1
Formaldehyde	H301, H311, H314, H317, H330, H331, H334, H335, H351	3, 8
Empty bottle contaminated by silane	H315	9
Empty jars	It is not possible to determine the hazards, as the contaminating chemical residues had not been specified	9
Methyl methacrylate	H225, H315, H317, H335	3
Methanol	H225, H301, H311, H331, H360, H370	3, 6.1
Mixture of xylenes	H226, H304, H312, H315, H332	3
Mixture: acetone with silane	H225, H319, H336	3
Aqueous solution: hydrogen peroxide and methanol	H225, H301, H311, H331, H360, H370	3, 6.1
Toluene	H225, H304, H315, H336, H361, H373	3

GHS: Globally Harmonized System of Classification and Labelling of Chemicals (GHS); *ANTT: Agência Nacional de Transportes* (National Transport Agency). Source: Prepared by the authors, based on ABNT^4^, Brazil^11^ and ECHA^15^.

Of the 14 chemical wastes inventoried, 13 (93%) had some hazard code. Empty bottles could not be classified due to the non-specification of the contaminating substances or mixtures (Table 1).

**Figure 2:**
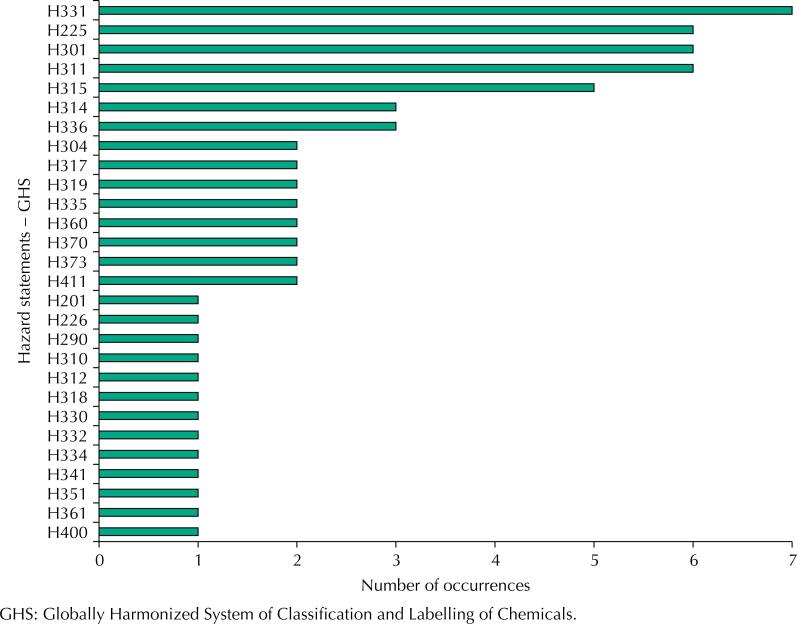
Occurrence of hazard codes and statements in FMUSP Laboratory I, from November 2017 to April 2019.

There were 28 types of hazards from these wastes, and the most frequent occurrences are shown in Figure 2. The most recurrent hazards were: H331 (toxic if inhaled) - 7 occurrences; H225 (highly flammable liquid and vapors) – 6; H301 (toxic if ingested) – 6; H311 (toxic in contact with the skin) – 6; H315 (causes irritation to the skin) – 5. The most frequent hazard in the laboratory is registered under code H331, the description of which is embodied in the properties of chemical residues of hydrochloric acid, picric acid, beta-mercaptoethanol (2-mercaptoethanol), phenol, formaldehyde, methanol and an aqueous solution of hydrogen peroxide and methanol.

The classification for external transport indicated that, of the 14 chemical wastes inventoried, all are considered hazardous (they have a hazard class or subclass).

In the inventory studied, chemical residues were found that have more than one risk class or subclass, represented by formaldehyde (3, 8), methanol (3, 6.1) and aqueous solution of hydrogen peroxide and methanol (3, 6.1). We found 17 occurrences concerning risk classes: risk class 3 (flammable liquids) - 8 occurrences; risk subclass 6.1 (toxic substances) – 4; risk class 9 (various hazardous substances and items) – 2; risk class 8 (corrosive substances) – 2; risk subclass 4.1 (flammable solids, self-reactive substances and desensitized solid explosives) - 1.

In Laboratory II, we found 26 types of chemical waste (Table 2), listed in alphabetical order and classified as the previous one (12 more items, compared to Laboratory I).

**Table 2:** Declared relationship of chemical residues generated in FMUSP Laboratory II, in the period from November 2017 to April 2019.

Chemical residue composition	Hazard statements (GHS codes)	Class or subclass of risk and subsidiary risk (ANTT)
Acetic acid	H314, H226	8
Azathioprine	H302, H315, H319, H335, H340, H350, H360, H361, H372	Substance or mixture not classified as hazardous for transport purposes
Dipyrone (Metamizole)	H302, H317, H334, H341, H351, H412	Substance or mixture not classified as hazardous for transport purposes
Class I filter for organic vapors (such as formaldehyde and methylamine), chlorine, chlorine dioxide, hydrofluoric acid, sulfur dioxide, hydrogen sulfide, ammonia and nitrogen dioxide	H331, H411	9
Agarose gel and tips with Sybr Safe® or Gel Red	It has no harmonized classification or risks notified by manufacturers, importers or users of this substance	Substance or mixture not classified as hazardous for transport purposes
Agarose gel and tips contaminated by Sybr Safe ® dye, red Gel or trizol (guanidine phenol isothiocyanate)	H301, H302, H311, H312, H314, H331, H332, H373, H411	6.1, 8
Sodium hydroxide	H290, H314	8
Leflunomide	H301, H315, H319, H335, H360, H361, H372	6.1
Lugol (aqueous solution of iodine and potassium iodide)	H312, H315, H317, H319, H332, H372, H400	8
Sodium mycophenolate	H302, H341, H360, H372, H410	9
Mixture: acetone, silane, formaldehyde, picric acid and acetic acid	H205, H225, H226, H301, H311, H314, H317, H319, H318, H330, H331, H334, H335, H336, H351	3, 8
Mixture: chloroform and phenol	H301, H302, H311, H314, H315, H319, H331, H341, H351, H361, H373, H411	6.1
Mixture: ethanol and formaldehyde	H225, H301, H311, H314, H317, H319, H330, H331, H334, H335, H351, H371	3, 8
Mixture: glutaraldehyde and silane	H226, H301, H314, H317, H330, H334, H335, H400, H411	8, 3
Mixture: methanol, bromophenol blue, glycine and tris (hydroxymethyl) aminomethane (TRIS)	H225, H301, H311, H312, H315, H319, H331, H332, H335, H360, H370	3, 6.1
Mixture: xylenes, ethanol, hydrogen peroxide, hematoxylin, 3,3' - diaminobenzidine and 3-amino-9-ethylcarbazole	H225, H226, H302, H304, H312, H315, H319, H332, H335, H336, 341, H350, H371	3, 6.1, 8
Mixture: xylenes, formaldehyde, ethanol, hydrogen peroxide, Fast Red, 3,3'-diaminobenzidine, 3-amino-9-ethylcarbazole, acetone, silane, picric acid and acetic acid	H225, H226, H301, H302, H304, H311, H312, H314, H315, H317, H319, H330, H331, H332, H334, H335, H341, H350, H351	3, 6.1, 8
Mixture: xylenes, formaldehyde, methanol, ethanol, hydrogen peroxide, acetic acid, Fast Red, 3,3'-diaminobenzidine, 3-amino-9-ethylcarbazole and acetone	H226, H301, H302, H304, H311, H312, H314, H315, H317, H319, H330, H331, H332, H334, H335, H341, H350, H351, H360, H370	3, 6.1, 8
N, n'-methylenebisacrylamide	H301, H302, H317, H318, H332, H340, H351, H360, H361, H372	6.1
Paracetamol	H302, H315, H317, H319, H332, H335, H341, H370, H371, H372, H373, H412	Substance or mixture not classified as hazardous for transport purposes
Prednisone	H312, H315, H319, H332, H360, H361, H373	Substance or mixture not classified as hazardous for transport purposes
Dowex ion exchange resin	H315, H318, H319, H335	Substance or mixture not classified as hazardous for transport purposes
5-bromo-2’-deoxyuridine (BrdU)solution	H315, H319, H335, H340, H341, H361	Substance or mixture not classified as hazardous for transport purposes
Fuchsin solution with phenol	H301, H302, H311, H314, H331, H341, H351, H373, H411	6.1
Buffer with beta-mercaptoethanol	H301, H310, H311, H315, H318, H331, H400, H411	6.1
Buffer with methanol	H225, H301, H311, H331, H360, H370	3, 6.1

GHS: Globally Harmonized System of Classification and Labelling of Chemicals (GHS); ANTT: *Agência Nacional de Transportes* (National Transport Agency). Source: Prepared by the authors, based on ABNT^4^, Brazil^11^ and ECHA^15^.

Of the 26 chemical wastes inventoried, 25 (96%) have some hazard code. However, an item had no harmonized classification or risk notified by the manufacturer, importer or user (Table 2).

**Figure 3:**
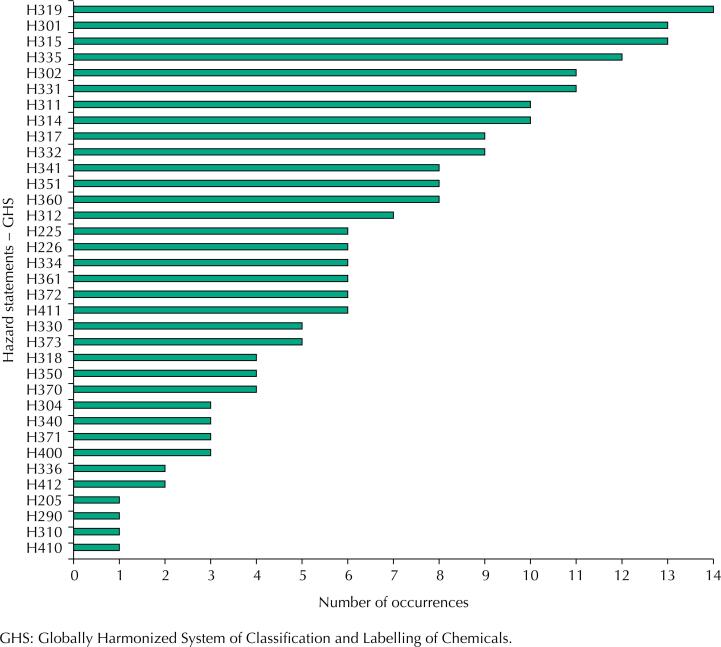
Occurrence of hazard codes and statements in FMUSP Laboratory II, from November 2017 to April 2019.

We found 35 types of hazards from these wastes, the most frequent occurrences are shown in Figure 3. Recurrent hazards in Laboratory II related to: H319 (causes severe eye irritation) – 14 cases; H301 (toxic if swallowed) – 13; H315 (causes irritation to the skin), – 13; H335 (may cause respiratory irritation) – 12; H302 (harmful if swallowed) – 11; H331 (toxic if inhaled) – 11; H311 (toxic in contact with skin) – 10; H314 (causes burns to severe skin and damage the eyes).

The classification for external transport showed that, of the 26 chemical wastes inventoried, 19 (73%) are considered hazardous and 7 (27%) are not classified as hazardous, thus do not have a risk class or UN number.

In the inventory of Laboratory II, we found chemical residues that have more than one risk class or subclass, such as a mixture of ethanol and formaldehyde (8, 3), a mixture of glutaraldehyde and silane (8, 3) and a buffer with methanol (3, 6.1). Of the 31 occurrences with the risk classes, they include: risk subclass 6.1 (toxic substances) – 11 occurrences; risk class 8 (corrosive substances) – 10 occurrences; risk class 3 (flammable liquids) – 8 occurrences; risk class 9 (miscellaneous hazardous substances and items) - 2 occurrences.

## DISCUSSION

Universities belong to a context of the plurality of areas of knowledge, the multiplicity of departments and sectors, heterogeneity of audience and courses, which contemplate several lines of research and contribute to the wide variety of inputs and reagents used in their laboratories. This scenario potentiates the generation of different and complex wastes, including chemicals, as well as may present a risk of exposure to the health and safety of workers, students and users.

The chemical safety agenda supported and disseminated by the *Fundação Jorge Duprat Figueiredo*, of Safety and Occupational Medicine (FUNDACENTRO), proposes promoting actions and discussions to adopt the GHS in universities, to attend undergraduate and postgraduate courses^16^ to promote health and reduce risks.

Quantification carried out in the practical classes of Inorganic Chemistry of the chemistry courses of the Universidade Federal Pelotas, identified about 130 substances used, originating more than 200 different preparations. Based on the GHS, the classification indicated that 90% of pure substances and 72% of preparations are considered dangerous in said system^17^.

In another university in the state of São Paulo, research developed with undergraduate Chemistry students indicated that GHS pictograms were not effective as a source of information about hazards associated with substances handled in the laboratory. Most students did not use chemical labels for this purpose, and students who used such instruments did not correctly identify the hazards represented by their symbols. Furthermore, the research pointed out that, in the field of GHS formulation, most students are unaware of the meaning of pictograms, since they are not self-explanatory^18^.

Therefore, GHS hazard statements, coupled with pictograms, are decisive for planning and implementing safety procedures relevant to chemical products and waste. Described on labels and sheets, the sentences allow the recognition of the potentially harmful effects that can affect users and the environment.

In our research, in the general inventory of chemical residues of the laboratories studied (I and II), representative of the academic environment of the higher education institution studied, we listed 40 items, of which 38 (95%) are classified as hazardous by the GHS. Of the 10 most frequent hazard codes and statements, nine are related to characteristics that confer risk to human health: H301 (19 occurrences), H315 (18), H331 (18), H311 (16), H319 (16), H335 (14), H314 (13), H302 (11) and H317 (11). Only code H225 (highly flammable liquid and vapors, 12 occurrences) represents a hazard framed as physical^6^. We highlight the occurrence of three codes linked to the toxicity of chemical residues by ingestion (H301), contact with the skin (H311) and/or inhalation (H331). Toxicity is the property that a substance has to produce an adverse effect on a living organism, as the result of the exposure^4^.

An important point found in both laboratories was the lack of specification of the conditions of some residues, concerning the concentration of the components, not making clear whether they included mixtures or pure substances. Since it is a retrospective inventory analysis, in the case of picric acid (Table 1), for example, the classification of greatest hazard was adopted, such as that defined for dry picric acid or with less than 30% water.

The scenario found confirmed the premise regarding the diversification and complexity of chemical waste generated in health education and research establishment, as well as the existence of several hazards to which users may be exposed. The results reinforce the requirement for the provision of collective protection equipment (CPE), personal protective equipment (PPE) and impact mitigation devices, including emergency showers, washers, fire extinguishers and absorbent and spill containment materials.

For the external transport stage, we found that the laboratories generated 33 (83%) types of chemical waste considered hazardous, mainly due to the characteristics of flammability (risk class 3, 16 occurrences), toxicity (risk subclass 6.1, 15 occurrences) and corrosivity (risk class 8, 12 occurrences). Only one listed chemical residue belongs to risk subclass 4.1 (picric acid), which is incompatible with any items in risk class 8^14^.

The need to offer institutional training programs to users, mandatory components of the implementation of institutional waste management plans is evident, as recommended by current legislation, which involves Federal Law No. 12,305/2010, RS No. 32 of the Ministry of Labor and Employment and Resolution of Collegiate Board No. 222/2018 of the *Agência Nacional de Vigilância Sanitária*^12,19,20^.

In the case of educational and research institutions in the field of Health, the training of students, technicians and researchers regarding the risks that chemical substances and their residues can present, is a primary and challenging task. Many professionals who work in these establishments have training in biology, biomedicine or medicine and generally have elementary knowledge about the physicochemical aspects and the reactivity of different substances, as well as the risks associated with the handling and generation of mixtures.

## CONCLUSION

In the laboratory environment and external transport, we found that the chemical residues generated in the studied sector are diversified in hazard characteristics, implying the possibility of varied and, in some cases, severe correlated risks, to which workers, students and the environment may be exposed.

Considering that such hazards are also present in reagents and other raw materials that give rise to chemical waste, laboratory activities require planning, techniques, regulations and special care regarding the management of stocks of substances and mixtures in all phases of the productive cycle of teaching and research.

GHS is a fundamental tool for accurate identification of chemical residues, inherent hazards and precautions in the work and external environments. In future work, this study may be supplemented by the inclusion of precautionary statements, which show recommended measures to be taken to prevent and mitigate adverse effects resulting from exposure, improper storage or handling of hazardous products and waste.

The use of hazardous goods transport legislation, specifically ANTT Resolution No. 5,947/2021, is essential for managing chemical waste in the steps outside the generating establishment, contributing to the minimization of transport risks.

It is understood that the tools used in this study are subject to extrapolation to other educational and research institutions, and this is a matter to be considered for adequacy of segregation and safe disposal of waste generated in similar institutions, in compliance with the legal requirements of waste management plans, with a view to sanitary, environmental and safety sustainability.
