# Supplementary material for: Laboratory chemical waste: hazard classification by GHS and transport risk
Source: Rev Saude Publica. 2021 Nov 23;55:102. doi: 10.11606/s1518-8787.2021055003259 (PMC8621485; doi:10.11606/s1518-8787.2021055003259)
Supplement: Translation

# Translation: Resíduos químicos laboratoriais: classificação de perigo pelo GHS e risco no transporte

**Authors:** Cássio Giovanni, Fabio Luiz Navarro Marques, Wanda Maria Risso Günther

**Affiliations:** I São Paulo SP Brasil Universidade de São Paulo. Faculdade de Saúde Pública. Doutorado em Saúde Global e Sustentabilidade. São Paulo, SP, Brasil; II São Paulo SP Brasil Universidade de São Paulo. Faculdade de Medicina. Departamento de Radiologia. São Paulo, SP, Brasil; III São Paulo SP Brasil Universidade de São Paulo. Faculdade de Saúde Pública. Departamento de Saúde Ambiental. São Paulo, SP, Brasil

**Keywords:** Resíduos Químicos, Resíduos Perigosos, Rotulagem de Produtos, Transporte de Substâncias, Produtos e Materiais

## Abstract

**OBJETIVOS:**

Identificar e avaliar, com base no Sistema Globalmente Harmonizado de Classificação e Rotulagem de Produtos Químicos (GHS) e na legislação da Agência Nacional de Transportes Terrestres (ANTT), os perigos provenientes dos resíduos químicos gerados em laboratórios de pesquisa na área de saúde.

**MÉTODOS:**

Resíduos químicos gerados em dois Laboratórios de Investigação Médica da Faculdade de Medicina da Universidade de São Paulo foram inventariados, no período de novembro de 2017 a abril de 2019, e classificados conforme o GHS (frases de perigo) e a legislação de transportes da ANTT (classes de risco), para determinação dos perigos provenientes das respectivas substâncias e misturas.

**RESULTADOS:**

No total, foram identificadas 40 substâncias ou misturas, cuja classificação pelo GHS indicou 36 frases de perigo, sendo 27 relacionadas à saúde humana. De acordo com a legislação estabelecida pela ANTT, foram encontrados 16 casos de periculosidade associada à inflamabilidade, 15 casos relacionados à toxicidade e 12 casos relativos à corrosividade.

**CONCLUSÕES:**

Resíduos químicos gerados nos laboratórios estudados são diversificados quanto a suas características de periculosidade, implicando a possibilidade de exposição a riscos severos aos trabalhadores, aos estudantes e ao ambiente. A correta identificação desses resíduos é fator primordial para diminuição da exposição aos riscos.

## INTRODUÇÃO

No contexto contemporâneo dos processos produtivos e do modelo econômico, com significativa ampliação do consumo de produtos químicos, maior celeridade na síntese de novos compostos e aumento da extração de matérias-primas, a geração de resíduos de diversas categorias é potencializada.

Produtos e resíduos químicos no meio ambiente podem afetar espécies e ecossistemas e até causar problemas em larga escala, como a depleção do ozônio estratosférico^1^. Compostos químicos, caso sejam utilizados e/ou descartados em desacordo com as diretrizes técnicas, científicas e legais, podem alterar as características e a qualidade dos compartimentos ambientais (ar, água, solo e biota), além de causar danos à saúde, à integridade física e às atividades do ser humano.

O desconhecimento acerca das propriedades e da periculosidade de produtos e resíduos químicos pode, inclusive, acarretar prejuízos ocupacionais aos profissionais envolvidos na manipulação e/ou no gerenciamento de tais substâncias, misturas ou materiais. É imprescindível que trabalhadores e estudantes implicados nessas atividades sejam capacitados e orientados sobre os riscos associados aos produtos químicos, assim como àqueles advindos do gerenciamento inadequado dos resíduos gerados.

Um aspecto fundamental nesse processo é a criação do Sistema Globalmente Harmonizado de Classificação e Rotulagem de Produtos Químicos (GHS), adotado formalmente em julho de 2003 pelo Comitê Econômico e Social das Nações Unidas^2^. Trata-se de sistema que promove, de forma padronizada e harmonizada, a identificação e a classificação de produtos químicos perigosos, servindo-se de frases e pictogramas afixados nos rótulos das embalagens e informados em fichas de segurança sobre a substância, a mistura ou o material em questão.

No Brasil, a classificação e a rotulagem preventiva de produtos químicos por meio do GHS têm caráter obrigatório, uma vez que a adoção do sistema está prevista na Norma Regulamentadora (NR) n° 26 – Sinalização de Segurança, desde que foi atualizada pela Portaria n° 229/2011, da Secretaria de Inspeção do Trabalho. Além disso, essa atualização estabelece que a estrutura e o conteúdo da ficha com dados de segurança do produto químico devem seguir as diretrizes do GHS^3^. Salienta-se ainda que a aplicação do GHS para fins de classificação, rotulagem e fichas de dados de segurança de produtos químicos é orientada pela Norma Brasileira (NBR) 14.725, partes 1, 2, 3 e 4, da Associação Brasileira de Normas Técnicas (ABNT)^4–7^.

Uma das disposições do GHS consiste nas frases de perigo, as quais dizem respeito a textos padronizados que descrevem a natureza e, se apropriado, a severidade do perigo^7–9^. A configuração das frases de perigo, indicadas pela letra H (*hazard*), está explicitada na Figura 1.

**Figura 1:** Especificação das frases de perigo (H), conforme GHS.

Fontes: elaborado pelos autores, com base em ABNT^4^, UNECE^8^ e Wallau e Santos^9^.

Ressalta-se ainda que o GHS propõe outros elementos para a comunicação do perigo, como a rotulagem, as frases de precaução e as fichas de dados de segurança^2,4,5^.

Como instituições públicas e empresas privadas brasileiras devem atender às exigências quanto à aplicação e aos preceitos do GHS, para que sejam asseguradas e aperfeiçoadas as condições de segurança laboral em suas instalações, torna-se fundamental a capacitação de trabalhadores e estudantes, com o propósito de eliminar ou mitigar impactos ambientais, à saúde e à segurança laboral.

Na perspectiva do transporte externo, produtos e resíduos perigosos, sejam de origem química, biológica ou radiológica, representam risco à vida, à saúde, ao ambiente e à segurança pública^10^. Para a identificação do perigo no transporte de cargas perigosas, foram definidas nove classes de risco expressas por números padronizados internacionalmente e definidas pela Resolução n° 5.947/2021, da Agência Nacional de Transportes Terrestres (ANTT)^11^.

As classes de risco, seus respectivos pictogramas e os códigos de identificação (números ONU) representam linguagem universal para o reconhecimento de produtos e resíduos perigosos transportados e, portanto, são fundamentais para a tomada de providências em caso de acidentes, vazamentos ou derramamentos, ou mesmo para o transporte transfronteiriço internacional.

Considerando-se que, em geral, os resíduos químicos oriundos de segmentos diversos como indústria, comércio, serviços de saúde, ensino e pesquisa são tratados fora das unidades geradoras, seu gerenciamento contempla também etapas e procedimentos externos, como o transporte até o tratamento ou a disposição final. Nesse caso, segundo as Leis Federais n° 12.305/2010 (Artigos 20 e 27) e n° 9.605/1998 (Artigos 2, 3, 54 e 56), o estabelecimento gerador é corresponsável (juntamente com o agente transportador) por classificar adequadamente os resíduos químicos no escopo do deslocamento de cargas perigosas, de acordo com as classes de risco^12,13^.

Na identificação e rotulagem, o conhecimento das informações sobre as classes de risco e respectiva legislação torna-se imprescindível para o correto preenchimento do Manifesto de Transporte de Resíduos (MTR), documento obrigatório na etapa de movimentação de cargas perigosas. A classe ou a subclasse de risco e, se existente, risco subsidiário são primordiais para a definição das devidas incompatibilidades entre os resíduos químicos, conforme ABNT NBR 14.619/2021^14^.

A organização de tais resíduos por meio do GHS e do sistema de transportes possibilita uma compreensão sistêmica dos riscos químicos associados. Tais riscos são considerados sob a ótica interna e externa da instituição e do ambiente laboratorial gerador, de forma contínua e complementar, levando em conta todo o processo de gerenciamento dos resíduos químicos gerados.

Pondera-se também que muitas substâncias podem ser consideradas perigosas nos parâmetros do GHS e/ou da ANTT, embora ainda não tenham sido estudadas e classificadas quanto a potenciais perigos, especialmente sobre toxicidade, mutagenicidade, carcinogenicidade e ecotoxicidade. Portanto, ao se tratar de tais substâncias, é preciso adotar o princípio da precaução, para que sejam salvaguardadas a saúde humana e a salubridade ambiental.

Os objetivos deste artigo são identificar e avaliar, com base no GHS (frases de perigo) e na legislação de transportes da ANTT (classes de risco), os resíduos químicos gerados em laboratórios de pesquisa na área de saúde, contribuindo para a melhoria do gerenciamento de resíduos químicos e de riscos e para a adoção de medidas de precaução, prevenção e contingência. Este trabalho poderá ser utilizado como apoio para implementação de política de qualidade em instituições similares à estudada, disseminando a todos os laboratórios e unidades as bases para atender à legislação pertinente ao gerenciamento de resíduos químicos.

## MÉTODOS

A pesquisa foi realizada na Faculdade de Medicina da Universidade de São Paulo (FMUSP), em dois Laboratórios de Investigação Médica (LIM), cujos resíduos químicos gerados são variados e complexos, muitos dos quais consistem em misturas de substâncias. Como fonte de dados, foi realizada análise retrospectiva dos resíduos químicos gerados e declarados pelos laboratórios em fichas de inventário, no período de novembro de 2017 a abril de 2019. Essas fichas são utilizadas para documentar o procedimento de encaminhamento dos resíduos químicos dos laboratórios ao abrigo (armazenamento externo) da instituição.

Para facilitar e unificar a compreensão e a identificação de cada resíduo químico, a terminologia dos itens listados pelos laboratórios foi padronizada de acordo com a nomenclatura técnica e/ou oficial das substâncias. Na etapa de determinação dos perigos decorrentes dos resíduos químicos gerados, foram considerados, conjuntamente, o âmbito laboratorial (GHS, ABNT NBR 14.725-3/2017) e a perspectiva do transporte externo (Resolução ANTT n° 5.947/2021).

Em se tratando do GHS, frases padronizadas de perigo (ABNT NBR 14.725-3/2017) foram atribuídas aos resíduos químicos, a fim de identificar características intrínsecas de periculosidade das substâncias presentes. Tais frases (H – *hazard*) são representadas por códigos e fazem parte da formulação e da aplicação do GHS^6^. Para cada substância componente do resíduo químico, foram obtidas as frases de perigo por meio de pesquisa no portal da Agência Europeia de Substâncias Químicas (ECHA)^15^.

Na classificação de embalagens vazias e demais objetos que tiveram contato com produtos químicos, foram consideradas as características das substâncias ou misturas contaminantes. Para os resíduos que continham misturas de substâncias oxidantes e inflamáveis, como peróxido de hidrogênio e metanol, respectivamente, foram empregados códigos e frases de perigo dos compostos inflamáveis, devido à baixa concentração dos oxidantes verificada.

Quanto ao transporte, os resíduos químicos foram classificados por meio de sua classe ou subclasse de risco e risco subsidiário, caso existente.

## RESULTADOS

No Laboratório I, foram encontrados 14 tipos de resíduos químicos (Quadro 1), listados em ordem alfabética e classificados conforme o GHS, com base na ABNT NBR 14.725-3/2017 e no portal da ECHA, e a Resolução ANTT n° 5.947/2021 (transporte de cargas perigosas).

**Quadro 1:** Relação declarada de resíduos químicos gerados no Laboratório I da FMUSP, no período de novembro de 2017 a abril de 2019.

Composição do resíduo químico	Frases de perigo (códigos do GHS)	Classe ou subclasse de risco e risco subsidiário (ANTT)
Acetona	H225, H319, H336	3
Ácido clorídrico, solução	H290, H314, H331	8
Ácido pícrico	H201, H301, H311, H331	4.1
Beta-mercaptoetanol	H301, H310, H311, H315, H318, H331, H400, H411	6.1
Fenol	H301, H311, H314, H331, H341, H373, H411	6.1
Formaldeído	H301, H311, H314, H317, H330, H331, H334, H335, H351	3, 8
Frasco vazio contaminado por silano	H315	9
Frascos vazios	Não é possível a determinação dos perigos, pois não foram especificados os resíduos químicos contaminantes	9
Metacrilato de metila	H225, H315, H317, H335	3
Metanol	H225, H301, H311, H331, H360, H370	3, 6.1
Mistura de xilenos	H226, H304, H312, H315, H332	3
Mistura: acetona com silano	H225, H319, H336	3
Solução aquosa: peróxido de hidrogênio e metanol	H225, H301, H311, H331, H360, H370	3, 6.1
Tolueno	H225, H304, H315, H336, H361, H373	3

GHS: Sistema Globalmente Harmonizado de Classificação e Rotulagem de Produtos Químicos; ANTT: Agência Nacional de Transportes. Fonte: elaborado pelos autores, com base em ABNT^4^, Brasil^11^ e ECHA^15^.

Dos 14 resíduos químicos inventariados, 13 (93%) possuem algum código de perigo. Os frascos vazios não puderam ser classificados, em decorrência da não especificação das substâncias ou misturas contaminantes (Quadro 1).

Foram verificados 28 tipos de perigos oriundos desses resíduos, cujas ocorrências mais frequentes encontram-se na Figura 2. Os perigos mais recorrentes foram: H331 (tóxico se inalado) – 7 ocorrências; H225 (líquido e vapores altamente inflamáveis) – 6; H301 (tóxico se ingerido) – 6; H311 (tóxico em contato com a pele) – 6; H315 (provoca irritação à pele) – 5. O perigo mais frequente no laboratório está registrado sob o código H331, cuja descrição se incorpora às propriedades dos resíduos químicos de ácido clorídrico, ácido pícrico, beta-mercaptoetanol (2-mercaptoetanol), fenol, formaldeído, metanol e solução aquosa de peróxido de hidrogênio e metanol.

**Figura 2:** Ocorrência dos códigos e frases de perigo no Laboratório I da FMUSP, no período de novembro de 2017 a abril de 2019.

A classificação para transporte externo indicou que, dos 14 resíduos químicos inventariados, todos são considerados perigosos (possuem classe ou subclasse de risco).

No inventário estudado, foram encontrados resíduos químicos que possuem mais de uma classe ou subclasse de risco, representados pelo formaldeído (3, 8), metanol (3, 6.1) e solução aquosa de peróxido de hidrogênio e metanol (3, 6.1). Foram encontradas 17 ocorrências em relação às classes de risco: classe de risco 3 (líquidos inflamáveis) – 8 ocorrências; subclasse de risco 6.1 (substâncias tóxicas) – 4; classe de risco 9 (substâncias e artigos perigosos diversos) – 2; classe de risco 8 (substâncias corrosivas) – 2; subclasse de risco 4.1 (sólidos inflamáveis, substâncias autorreagentes e explosivos sólidos insensibilizados) – 1.

No Laboratório II, foram encontrados 26 tipos de resíduos químicos (Quadro 2), listados em ordem alfabética e classificados como o anterior (12 itens a mais, em comparação com o Laboratório I).

**Quadro 2:** Relação declarada de resíduos químicos gerados no Laboratório II da FMUSP, no período de novembro de 2017 a abril de 2019.

Composição do resíduo químico	Frases de perigo (códigos do GHS)	Classe ou subclasse de risco e risco subsidiário (ANTT)
Ácido acético	H314, H226	8
Azatioprina	H302, H315, H319, H335, H340, H350, H360, H361, H372	Substância ou mistura não classificada como perigosa para fins de transporte
Dipirona (metamizol)	H302, H317, H334, H341, H351, H412	Substância ou mistura não classificada como perigosa para fins de transporte
Filtro classe I para vapores orgânicos (como formaldeído e metilamina), cloro, dióxido de cloro, ácido fluorídrico, dióxido de enxofre, sulfeto de hidrogênio, amônia e dióxido de nitrogênio	H331, H411	9
Gel de agarose e ponteiras com Sybr Safe® ou Gel Red	Não possui classificação harmonizada nem riscos notificados pelos fabricantes, importadores ou usuários desta substância	Substância ou mistura não classificada como perigosa para fins de transporte
Gel de agarose e ponteiras contaminadas pelo corante Sybr Safe®, por Gel Red ou por trizol (isotiocianato de guanidina e fenol)	H301, H302, H311, H312, H314, H331, H332, H373, H411	6.1, 8
Hidróxido de sódio	H290, H314	8
Leflunomida	H301, H315, H319, H335, H360, H361, H372	6.1
Lugol (solução aquosa de iodo e iodeto de potássio)	H312, H315, H317, H319, H332, H372, H400	8
Micofenolato de sódio	H302, H341, H360, H372, H410	9
Mistura: acetona, silano, formaldeído, ácido pícrico e ácido acético	H205, H225, H226, H301, H311, H314, H317, H319, H318, H330, H331, H334, H335, H336, H351	3, 8
Mistura: clorofórmio e fenol	H301, H302, H311, H314, H315, H319, H331, H341, H351, H361, H373, H411	6.1
Mistura: etanol e formaldeído	H225, H301, H311, H314, H317, H319, H330, H331, H334, H335, H351, H371	3, 8
Mistura: glutaraldeído e silano	H226, H301, H314, H317, H330, H334, H335, H400, H411	8, 3
Mistura: metanol, azul de bromofenol, glicina e tris (hidroximetil) aminometano (TRIS)	H225, H301, H311, H312, H315, H319, H331, H332, H335, H360, H370	3, 6.1
Mistura: xilenos, etanol, peróxido de hidrogênio, hematoxilina, 3,3’-diaminobenzidina e 3-amino-9-etilcarbazol	H225, H226, H302, H304, H312, H315, H319, H332, H335, H336, 341, H350, H371	3, 6.1, 8
Mistura: xilenos, formaldeído, etanol, peróxido de hidrogênio, Fast Red, 3,3’-diaminobenzidina, 3-amino-9-etilcarbazol, acetona, silano, ácido pícrico e ácido acético	H225, H226, H301, H302, H304, H311, H312, H314, H315, H317, H319, H330, H331, H332, H334, H335, H341, H350, H351	3, 6.1, 8
Mistura: xilenos, formaldeído, metanol, etanol, peróxido de hidrogênio, ácido acético, Fast Red, 3,3’-diaminobenzidina, 3-amino-9-etilcarbazol e acetona	H226, H301, H302, H304, H311, H312, H314, H315, H317, H319, H330, H331, H332, H334, H335, H341, H350, H351, H360, H370	3, 6.1, 8
N, N’-metilenobisacrilamida	H301, H302, H317, H318, H332, H340, H351, H360, H361, H372	6.1
Paracetamol	H302, H315, H317, H319, H332, H335, H341, H370, H371, H372, H373, H412	Substância ou mistura não classificada como perigosa para fins de transporte
Prednisona	H312, H315, H319, H332, H360, H361, H373	Substância ou mistura não classificada como perigosa para fins de transporte
Resina de troca iônica Dowex	H315, H318, H319, H335	Substância ou mistura não classificada como perigosa para fins de transporte
Solução de 5-bromo-2’-deoxiuridina (BrdU)	H315, H319, H335, H340, H341, H361	Substância ou mistura não classificada como perigosa para fins de transporte
Solução de fucsina com fenol	H301, H302, H311, H314, H331, H341, H351, H373, H411	6.1
Tampão com beta-mercaptoetanol	H301, H310, H311, H315, H318, H331, H400, H411	6.1
Tampão com metanol	H225, H301, H311, H331, H360, H370	3, 6.1

GHS: Sistema Globalmente Harmonizado de Classificação e Rotulagem de Produtos Químicos; ANTT: Agência Nacional de Transportes. Fonte: elaborado pelos autores, com base em ABNT^4^, Brasil^11^ e ECHA^15^.

Dos 26 resíduos químicos inventariados, 25 (96%) possuem algum código de perigo. Verificou-se que um item não possuía classificação harmonizada nem riscos notificados pelo fabricante, importador ou usuário (Quadro 2).

Foram encontrados 35 tipos de perigo oriundos desses resíduos, cujas ocorrências mais frequentes são apresentadas na Figura 3. Os perigos recorrentes no Laboratório II referiam-se a: H319 (provoca irritação ocular grave) – 14 ocorrências; H301 (tóxico se ingerido) – 13; H315 (provoca irritação à pele) – 13; H335 (pode provocar irritação das vias respiratórias) – 12; H302 (nocivo se ingerido) – 11; H331 (tóxico se inalado) – 11; H311 (tóxico em contato com a pele) – 10; H314 (provoca queimadura severa à pele e dano aos olhos).

**Figura 3:** Ocorrência dos códigos e frases de perigo no Laboratório II da FMUSP, no período de novembro de 2017 a abril de 2019.

A classificação para transporte externo mostrou que, dos 26 resíduos químicos inventariados, 19 (73%) são considerados perigosos, ao passo que 7 (27%), por não serem classificados como perigosos, não possuem classe de risco nem número ONU.

No inventário do Laboratório II, foram encontrados resíduos químicos que possuem mais de uma classe ou subclasse de risco, como mistura de etanol e formaldeído (8, 3), mistura de glutaraldeído e silano (8, 3) e tampão com metanol (3, 6.1). Das 31 ocorrências em relação às classes de risco, incluem-se: subclasse de risco 6.1 (substâncias tóxicas) – 11 ocorrências; classe de risco 8 (substâncias corrosivas) – 10 ocorrências; classe de risco 3 (líquidos inflamáveis) – 8 ocorrências; classe de risco 9 (substâncias e artigos perigosos diversos) – 2 ocorrências.

## DISCUSSÃO

Universidades pertencem a um contexto de pluralidade de áreas de conhecimento, multiplicidade de departamentos e setores, heterogeneidade de público e de cursos, que contemplam diversas linhas de pesquisa e contribuem para a grande variedade de insumos e reagentes empregados em seus laboratórios. Esse cenário potencializa a geração de diferentes e complexos resíduos, incluindo os químicos, assim como pode apresentar risco de exposição à saúde e segurança dos trabalhadores, estudantes e usuários.

A agenda de segurança química respaldada e difundida pela Fundação Jorge Duprat Figueiredo, de Segurança e Medicina do Trabalho (FUNDACENTRO) propõe a promoção de ações e discussões no sentido de se adotar o GHS nas universidades, de forma a atender aos cursos de graduação e pós-graduação^16^, com o intuito de promoção da saúde e redução dos riscos.

Quantificação realizada nas aulas práticas de Química Inorgânica dos cursos de Química, da Universidade Federal de Pelotas, identificou cerca de 130 substâncias utilizadas, originando mais de 200 preparações diferentes. Com base no GHS, a classificação indicou que 90% das substâncias puras e 72% das preparações são consideradas perigosas no referido sistema^17^.

Em outra universidade do estado de São Paulo, pesquisa desenvolvida com estudantes de graduação em Química indicou que os pictogramas do GHS não foram eficazes como fonte de informação de perigos associados às substâncias manipuladas no laboratório. A maioria dos discentes não se valia dos rótulos dos produtos químicos para essa finalidade, e os alunos que usavam tais instrumentos não identificavam corretamente os perigos representados pelos respectivos símbolos. A pesquisa assinalou que, no campo da formulação do GHS, a maioria dos estudantes desconhece o significado dos pictogramas, pois estes não são autoexplicativos^18^.

Logo, as frases de perigo do GHS, atreladas aos pictogramas, são determinantes para o planejamento e a implantação de procedimentos de segurança pertinentes aos produtos e resíduos químicos. Dispostas nos rótulos e nas fichas, as frases permitem o reconhecimento dos potenciais efeitos deletérios que podem acometer usuários e o ambiente.

Em nossa pesquisa, no inventário geral de resíduos químicos dos laboratórios estudados (I e II), representativos do ambiente acadêmico da instituição de ensino superior estudada, foram listados 40 itens, dos quais 38 (95%) são classificados como perigosos pelo GHS. Dos 10 códigos e frases de perigo mais frequentes, nove estão relacionados a características que conferem risco à saúde humana: H301 (19 ocorrências), H315 (18), H331 (18), H311 (16), H319 (16), H335 (14), H314 (13), H302 (11) e H317 (11). Apenas o código H225 (líquido e vapores altamente inflamáveis, 12 ocorrências) representa um perigo enquadrado como físico^6^. Destaca-se a ocorrência de três códigos ligados à toxicidade dos resíduos químicos, por ingestão (H301), contato com a pele (H311) e/ou inalação (H331). Toxicidade é a propriedade que uma substância tem de produzir efeito adverso a um organismo vivo, em decorrência da exposição^4^.

Um importante ponto encontrado em ambos os laboratórios foi a falta de especificação das condições de alguns resíduos, com relação à concentração dos componentes, não deixando claro se compreendiam misturas ou substância pura. Por se tratar de análise retrospectiva de inventário, no caso do ácido pícrico (Quadro 1), por exemplo, foi adotada a classificação de maior perigo, como a definida para o ácido pícrico seco ou com menos de 30% de água.

O cenário encontrado confirmou a premissa quanto à diversificação e complexidade dos resíduos químicos gerados em estabelecimento de ensino e pesquisa em saúde, assim como a existência de diversos perigos a que os usuários podem estar expostos. Os resultados reforçam a exigência de provimento de equipamentos de proteção coletiva (EPC), equipamentos de proteção individual (EPI) e dispositivos de mitigação de impactos decorrentes de acidentes e incidentes nos laboratórios de instituições de ensino e pesquisa em saúde. No caso desses últimos, incluem-se chuveiros de emergência, lava-olhos, extintores e materiais absorventes e de contenção de derramamentos.

Para a etapa de transporte externo, constatou-se que os laboratórios geraram 33 (83%) tipos de resíduos químicos considerados perigosos, principalmente devido às características de inflamabilidade (classe de risco 3, 16 ocorrências), toxicidade (subclasse de risco 6.1, 15 ocorrências) e corrosividade (classe de risco 8, 12 ocorrências). Somente um resíduo químico listado pertence à subclasse de risco 4.1 (ácido pícrico), a qual é totalmente incompatível com quaisquer itens da classe de risco 8^14^.

Fica evidente a necessidade de oferta de programas institucionais de treinamento e capacitação aos usuários, componentes obrigatórios da implementação dos planos institucionais de gerenciamento de resíduos, conforme preconizado pela legislação vigente, que envolve a Lei Federal n° 12.305/2010, a NR n° 32 do Ministério do Trabalho e Emprego e a Resolução da Diretoria Colegiada n° 222/2018 da Agência Nacional de Vigilância Sanitária^12,19,20^.

No caso das instituições de ensino e pesquisa na área da saúde, a capacitação de alunos, técnicos e pesquisadores, com relação ao entendimento sobre os riscos que substâncias químicas e seus resíduos podem apresentar, é tarefa primordial e desafiadora. Muitos profissionais que atuam nesses estabelecimentos possuem formação nas áreas de biologia, biomedicina ou medicina e, geralmente, têm conhecimentos elementares sobre os aspectos físico-químicos e a reatividade de diferentes substâncias, assim como dos riscos associados à manipulação e geração de misturas.

## CONCLUSÃO

No ambiente laboratorial e no transporte externo, constatou-se que os resíduos químicos gerados no setor estudado são diversificados quanto às características de periculosidade, implicando a possibilidade de riscos correlatos variados e, em alguns casos, severos, aos quais trabalhadores, estudantes e o meio ambiente podem ser expostos.

Levando em conta que tais perigos estão presentes também nos reagentes e nas demais matérias-primas que dão origem aos resíduos químicos, as atividades laboratoriais requerem planejamento, técnicas, regramentos e cuidados especiais referentes ao gerenciamento dos estoques das substâncias e misturas, em todas as fases do ciclo produtivo de ensino e pesquisa.

O GHS consiste em ferramenta fundamental para identificação apurada dos resíduos químicos, dos perigos inerentes e das precauções a serem tomadas no ambiente de trabalho e no ambiente externo. Em trabalhos futuros, este estudo poderá ser complementado com a inclusão das frases de precaução, que apresentam medidas recomendadas a serem tomadas para prevenção e mitigação de efeitos adversos resultantes da exposição, da armazenagem inapropriada ou do manuseio de produtos e resíduos perigosos.

A utilização da legislação de transporte de cargas perigosas, especificamente a Resolução ANTT n° 5.947/2021, é imprescindível para o gerenciamento dos resíduos químicos nas etapas externas ao estabelecimento gerador, contribuindo para minimização de riscos de transporte.

Entende-se que as ferramentas empregadas neste estudo são passíveis de extrapolação para outras instituições de ensino e pesquisa, e este é um assunto a ser considerado para adequação da segregação e destinação seguras dos resíduos gerados em instituições similares, em atendimento aos requisitos legais dos planos de gerenciamento de resíduos, com vistas à sustentabilidade sanitária, ambiental e de segurança.
